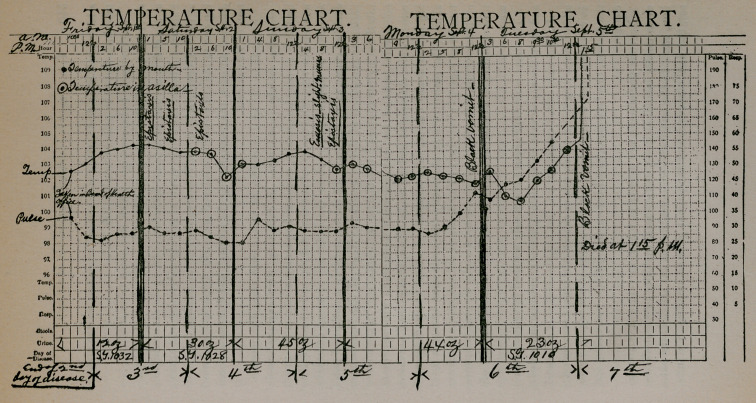# The Case of Yellow Fever Recently Occurring in Atlanta, Ga.

**Published:** 1905-11

**Authors:** William B. Summerall

**Affiliations:** Atlanta, Ga.


ATLANTA

### Journal-Record of Medicine

Successor to Atlanta Medical and Surgical Journal, Established 1855, and Southern Medical Record, Established 1870.

OWNED BY THE ATLANTA MEDICAL JOURNAL CO.

**Published Monthly.**

VOL. VII. NOVEMBER, 1905. No. 8

BERNARD WOLFF, M.D., EDITOR, Nos. 319–20 Prudential.

M. B. HUTCHINS, M.D., BUSINESS MANAGER, 1007–1008 Century Bldg.

E. G. BALLENGER, M.D., ASSOCIATE EDITOR AND ASSISTANT MANAGER.

J. N. LECONTE, M.D., FOREIGN CORRESPONDENT.

### *ORIGINAL COMMUNICATIONS.*

### THE CASE OF YELLOW FEVER RECENTLY OCCURRING IN ATLANTA, GA.

### HISTORY, CLINICAL NOTES AND OBSERVATIONS.

By WILLIAM B. SUMMERALL, M.D., ATLANTA.

J. C. C., mechanic by trade; age thirty-nine years; weight 182 pounds. Lived in infected district in Pensacola, Fla.

Left Pensacola, Fla., at noon, Wednesday, August 30, 1905, and arrived in Atlanta about 2 a. m., Thursday, August 31.

Was taken sick about the time he left Pensacola, noon, Wednesday. No history of chill or rigor, no vomiting, no nausea; had "severe headache and felt feverish." Remained in bed at his hotel in Atlanta from the time of his arrival until about 4 p. m., of the same day, when he went on the streets for about half an hour, then returned to his bed, which he kept until next morning, Friday, September 1st, when he reported to the Board of Health office.

It was in the Board of Health office that I first saw patient at 10:30 a. m., at which time the first recorded pulse and temperature were taken. At this time face was flushed, eyes intensely congested, respiration rapid, and an uneasy, restless condition was noted. The total ensemble of objective symptoms gave the impression that he was more ill than the answers to questions propounded would indicate. He had been walking the streets of the city just previously, and this fact must not be lost sight of in considering the first recorded pulse rate.

He was put to bed in the Isolation Hospital, a five-room cottage on the outskirts of the city, which I had especially prepared for such an emergency by direction of the health authorities, as soon as he could be driven there, I, myself, accompanying him. Complained of intense headache, only slight backache, and very little pain or ache in lower extremities. He had now been sick two days.

A *positive* diagnosis of yellow fever could and would have been made at this time, had the facilities for urinalysis been at hand. The fact of his being taken at once to the Isolation Hospital is sufficient evidence of a preliminary or "public safety" diagnosis having already been made—(I may state right here that, personally, I am not inclined to make a *positive* diagnosis of yellow fever without a show of albumin in the urine during the course of the disease. Preliminary diagnoses of yellow fever are frequently and usually made as a matter of "public protection" during epidemics in this country, and in what has been termed the "focal zone of infection," when dealing with fevers that can not readily be diagnosed otherwise. In general they are regarded as "suspicious," but it has always been my practice to *confirm* the diagnosis, if the case prove to be yellow fever, or revise it, if it prove otherwise, or modify it by relegating it to the not inconsiderable list of "doubtful" cases, if albumin is not found. Were such a policy strictly adhered to in this country during epidemics, wherein "public protection" diagnoses of yellow fever must be made in a large number of cases upon the first visit of the physician to a fever patient, such diagnoses being revised officially, and the patients themselves so notified, in those cases which clearly prove to be not yellow fever, and even admitting all cases that would be regarded as "doubtful" to the yellow fever list, we would come nearer getting at accurate statistics of mortality, and meet with fewer instances of so-called second attacks).

At 5:30 p. m. a *positive* diagnosis of yellow fever was made. Facilities for making the urinalysis were not procurable until this hour, but the urine passed at 11:30 a. m. and again at 2 p. m., was saved for analysis, and each specimen was loaded with albumin. The diagnosis was based upon the following symptoms, viz., the facies, which were typical; lack of correlation between pulse and temperature; capillary congestion; spongy condition of gums, and the albuminuria, which was excessive in amount as early as the end of the second day.

When the diagnosis was completed, the announcement of "a genuine, good case of yellow fever" was made to the City Health Officer as soon as he could be communicated with. It was then after dark, and as the Isolation Hospital was inconveniently located on the outskirts of the city, further consultation was deferred until the next (Saturday) morning. On Saturday morning the City Health Officer, Dr. J. P. Kennedy, came out early to see the case. He concurred in the diagnosis. While in consultation we were mutually of the opinion that as the announcement of a case of yellow fever in the city would probably disturb the equilibrium of Atlanta, and possibly of the State of Georgia, that further consultation and affirmation of the diagnosis would be a precautionary measure of value, and it was decided to call in Dr. Jno. C. Olmsted, who has had an extensive experience with the disease in former epidemics in Southern cities, and who has had charge, in more recent years, of a number of sporadic cases developing among refugees from infected localities, who sought safety in Atlanta. Dr. Olmsted visited the patient on Saturday morning, and at once concurred in the diagnosis. The City Health Officer then promptly reported the case to the Mayor, and the announcement at once was given to the newspapers.

Prognosis at this time was grave, and I so announced it to those in consultation, and to a number of other physicians who visited the case later in the day. A temperature averaging 104° throughout the third day, (it must be remembered that patient had been sick with the disease two days before being taken in charge); the unusually high percentage of albumin in the urine at the end of the second day, (and I have no hesitation in saying that it could probably have been found in abundance twenty-four hours earlier); and the tendency to hemorrhage, which was marked, form altogether about the most unfavorable combination of symptoms in yellow fever with which the physician can be confronted. My experience with the disease in its native zone, covering a period of about three years as a surgeon of volunteers with the United States army in Cuba, has taught me to be on the alert when either of the above symptoms is encountered in an exaggerated degree. The accentuation of either may be likened to the advance, in force, of either the center, or the right, or the left wing of an opposing army, whereas their combination in the same subject in an exaggerated degree is like the simultaneous advance of an entire army, center, right and left, in full force, with determination to sweep the entire field. The prognosis became more and more unfavorable each day and each hour, as there was no appreciable abatement of the grave symptoms above cited.

The amount of urine passed during the first fourteen hours was twelve ounces. A diminution of this amount would have rendered the case even more serious, while suppression, of course, is fatal. The kidneys must always be watched in this disease, as they are a point of great danger. Under the influence of mild diuretics the quantity of urine passed from midnight Friday to midnight Saturday was increased to thirty ounces, and the next twenty-four hours to forty-five ounces, and the next to forty-four ounces, and the last thirteen hours gave twenty-three ounces. The specific gravity of the first specimen examined was 1032, and this gradually decreased until the last specimen gave 1010. It was intensely highly colored at the beginning, but as the quantity increased, intensity of color diminished. There was no diminution in quantity of albumin.

The temperature continued abnormally high. In viewing the accompanying chart, bear in mind that half the records show axillary temperature. An hour before death, the thermometer showed 104° in the axilla. At the beginning of the post mortem, two and

TEMPERATURE CHART.

TEMPERATURE CHART.

Friday Sept. 1st

Saturday Sept. 2

Sunday Sept. 3

Monday Sept. 4

Tuesday Sept. 5th

A.M.

P.M.

Hour

Temp.

Pulse.

Resp.

• Temperature by mouth

⊙ Temperature in axilla

Temp

Pulse

Epistaxis

Epistaxis

Epistaxis

Emesis slight nausea

Epistaxis

Black vomit

Black vomit

Died at 1.15 P.M.

Stools.

Urine.

Day of Disease.

12 oz

S.G. 1032

30 oz

S.G. 1028

45 oz

44 oz

23 oz

S.G. 1010

End of 2nd day of disease

3rd

4th

5th

6th

7th a half hours after death, the thermometer immersed in the abdomen just above the pelvis recorded 105 3-5°.

The tendency to hemorrhage, which was noted from the first observations, was further accentuated by free epistaxis at 1 o'clock a. m., at 5 a. m. and at 2 p. m. Saturday, and again at midnight Sunday. There was black vomit at midnight Monday and again at time of death, 1:15 p. m. Tuesday, the last mechanical only being due to the last powerful contractions of the diaphragm and abdominal muscles, thus compressing the stomach and ejecting a portion of its contents.

At no time during the course of the disease were the gastric symptoms unduly prominent. In fact, they were overshadowed by those already mentioned, and are frequently much more pronounced in very mild cases. At no time did the patient especially complain of nausea, except under the influence of some external exciting cause, as for instance, the thermometer in mouth, and palpation or percussion of the epigastrium. None of the liquids imbibed during the course of the disease were rejected by the stomach—water, crushed ice and lemonade were taken with apparent relish, while champagne and other alcoholics, though not provoking nausea to any extent, were complained of as being disagreeable. (The patient was an absolutely temperate man, in fact, a teetotalist as regards alcoholics). The first and only emesis, exclusive of black vomit, followed the last attempt to take an oral temperature at midnight Sunday, and consisted of only a small quantity of mucous.

The heart is another organ to be closely watched in yellow fever, and upon the appearance of signals of distress it must be supported.

In the case under consideration, the tongue, which is not usually of much diagnostic value, was as characteristic as is ever found. It was clean, red, congested. If it was coated at the beginning of the disease, this had disappeared before the patient was taken in charge.

Nervous phenomena are usually met with in yellow fever, such as an uneasy mental attitude, restlessness, inability to sleep, delirium, etc. In the case under consideration, as was stated at the outset, the first observation of the patient gave an impression of uneasiness or mental apprehension, not nearly so well marked, however, as is frequently met with in those who labor under excitement when in a focus of infection. Only at intervals was the patient restless and unable to sleep. It has been my experience that these conditions are most exaggerated in those cases wherein the gastric symptoms are most pronounced. Twenty-four hours before death the patient became slightly delirious, and this condition gradually increased to the end, with occasional lucid intervals. The delirium was of a mild, busy type. At no time was there any inclination to be violent,

As to the jaundice, the "yellow" of yellow fever, there is usually either surprise or disappointment in store for those who see the disease in the living for the first time. This is true, as I have often seen demonstrated, of many members of the profession, as well as of laymen. The "yellow" is the most impressive part of the nomenclature, and it occupies the place of prominence in the mental picture. In the case under consideration, when the patient was first seen and taken in charge, the jaundice was very slight and hardly capable of appreciation by those not familiar with the disease. Of the several physicians who visited the case on Saturday and Sunday, the second and third days after he had been taken in charge, the same being the fourth and fifth days of the disease, there were a few who expressed to me their surprise at not seeing a more pronounced jaundice. In many mild cases of the disease the jaundice plays a minor role and is practically inappreciable, while in more pronounced cases, if the patient live long enough, the jaundice becomes more manifest during life, and in all cases terminating fatally, it reaches its maximum after death. In the case under consideration the icterns gradually increased during life, and after death the body presented the characteristic saffron hue. It affects alike all the tissues of the body, but in the fats it shows probably to the greatest advantage.

As to treatment for yellow fever, I know of no particular or specific course of medication that can be regarded as such. It must be symptomatic, meeting conditions as they are anticipated and as they arise. Some cases have individual characteristics which influence certain procedures. For instance, I have had some patients with yellow fever, who felt comfortable only when wrapped in blankets and with hot water bottles in the bed, and this in the tropics during the heat of summer. (Such was my own individual peculiarity when stricken with the disease). And this was regardless of the course of temperature, whether high or low. Others could not tolerate such a procedure, and if it were attempted, the moment the surgeon's or the nurse's back was turned, blankets and hot water bags would be discarded in spite of injunctions to the contrary. In this matter I am disposed to consult the patient's individual comfort and act accordingly. I can not say that I have observed any marked beneficial results on temperature of this disease from profuse diaphoresis. In the case under consideration, a gentle diaphoresis was maintained practically throughout the course of the disease. At 2 p. m., Friday, it being observed that the temperature of the patient was steadily climbing, a 15 gr. dose of quinine was administered, tentatively, to determine whether or not there was a malarial element involved. As will be seen by the chart, the temperature continued to climb, although there was profuse diaphoresis during the latter part of the afternoon. Alcohol sponges were also employed with the object of reducing temperature. Hot water bottles to the feet and ice cap to the head alleviated the intense headache of which the patient complained.

As has been noted, the kidneys must be watched, and a free and copious flow of urine maintained if possible. The results obtained in this case as noted above, were fairly satisfactory.

For nausea and vomiting individual cases will respond to specia treatment. As simple remedies as lemon or limeade will be satisfactory in some cases. Effervescent drinks will be grateful to many. Champagne will give good results in some cases while in others it will not. In the more aggravated conditions my personal experience has been that cocaine administered by the mouth gives the greatest percentage of good results. In connection with this I have usually administered chloral hydrate and sodium bromide, thereby meeting the restless condition that accompanies the excessive nausea and vomiting, as noted previously. If sleep can be procured the stomach is quieted, or if the stomach is quited sleep can be induced. Morphia handled with care can be used in selected cases. In the case under consideration chloral hydrate was used successfully to control the occasional manifestations of restlessness.

The gastric hemorrhage producing the black vomit of yellow fever is, of course, a very grave symptom, yet it is by no means an unfailing sign of approaching dissolution. Those who have an extensive experience with this disease, will occasionally be most agreeably surprised by seeing a most desperate and apparently hopeless case of this kind progress to recovery. I have seen a few such recoveries, my own individual case, in which the black vomit was as copious and persistent for two days, as I ever unwillingly witnessed, being one of them. Of course there are cases, fatal and otherwise, in which the phenomenon of black vomit does not appear externally as such, although there is gastric hemorrhage. The hemorrhage is not expelled from the stomach. A peculiarity of this hemorrhage is the manner in which it is produced. In ordinary hemorrhage there is usually visible a solution of continuity at the bleeding point, whereas, in the gastric hemorrhage of yellow fever a most critical examination of the mucous membrane of the stomach will frequently fail to reveal such a lesion. In such cases it is not in reality a hemorrhage, that is a *flowing* of blood, but rather an *oozing* of blood. I have seen this take place from the mouth, the nose, the eyes, and have held the eye open and watched it during the process without being able to detect any lesion. It comes as does the sweat upon the brow. I have used many different styptics in the effort to control this hemorrhage, but in so far as ultimate benefit to the patient is concerned, I have not one to especially recommend. In no case coming within my own observation, have I regarded the black vomit (gastric hemorrhage) as being the cause of death, even remotely, but rather as a result of other malignant forces at work, resulting in extensive disintegration of the blood, and deterioration of the integrity of the walls of the blood vessels themselves.

I was present at the post mortem held in the above case by Dr. H. F. Harris, and the usual typical findings in yellow fever were present. Among these were the rigor mortis, which comes on rapidly, and the deep jaundice accentuated after death. The stomach contained several ounces of black vomit; the duodenum stained with the same. The kidneys were friable, due to granular degeneration. The brain and its membranes were congested.

To recapitulate, will state that the case from the first was typical, practically all of the diagnostic symptoms, both objective and subjective, being unusually well marked.

Prognosis from the first was very grave, and I so announced it officially to the proper health authorities. With each hour and each day it became more serious, so much so, in fact, that on Sunday, and again on Monday, I hazarded the prediction that the patient would not survive longer than sunrise Tuesday morning. As an actual fact, he lived until 1:15 p. m., of the same day, although on the verge of dissolution for many hours previous. I repeat these statements here, because of the fact that the newspaper accounts were of the most favorable nature, beginning with the statement that there was no doubt but that the patient would recover, and continuing their "doing well" accounts even after I had set, and so stated officially, the hour beyond which I did not expect him to survive. It must not be understood that I hereby accuse or remotely imply that the newspapers, or any of their representatives, misquoted me, or misrepresented my views in regard to the case, for as a fact at no time did I personally or by authorized substitute, hold any communication of any kind with any newspaper or newspaper representative. I had no opportunity of doing so. I did not see the published statements until after the demise of the patient, else I would have stated the facts officially to the papers, and I have no doubt they would have been published as stated.

I wish to pay a tribute, a deserved tribute, to Miss Leila Hunt, the trained nurse, who came to my rescue on Saturday afternoon and remained with the patient to the end. It was her first experience with yellow fever, and she proved herself both brave and capable.

I am also indebted to Dr. Jno. C. Olmsted for consultations in the case.

The State Board of Health, then in session in Atlanta, visited the patient in a body on Saturday afternoon. The case was also visited by about twenty of the local physicians during the four days of illness.

## Figures and Tables

**Figure f1:**